# Reliability of cancer screening questions from the National Health Interview Survey

**DOI:** 10.1371/journal.pone.0352356

**Published:** 2026-07-01

**Authors:** Larry G. Kessler, Bryan Comstock, Erin J. Aiello Bowles, Jin Mou, Michael G. Nash, Perla Bravo, Lynn E. Fleckenstein, Chaya Pflugeisen, Hongyuan Gao, Rachel L. Winer, India J. Ornelas, Cynthia Smith, Christine Neslund-Dudas, Punith Shetty, Uma G. Raghavan

**Affiliations:** 1 Department of Health Systems and Population Health, University of Washington School of Public Health, University of Washington, Seattle, Washington, United States of America; 2 Department of Biostatistics, University of Washington School of Public Health, University of Washington, Seattle, Washington, United States of America; 3 Kaiser Permanente Washington Health Research Institute, Kaiser Permanente Washington, Seattle, Washington, United States of America; 4 Institute for Research and Innovation, MultiCare Health System, Tacoma, Washington, United State of America; 5 Department of Epidemiology, University of Washington School of Public Health, University of Washington, Seattle, Washington, United States of America; 6 Department of Public Health Sciences, Henry Ford Health, Detroit, Michigan, United States of America; 7 Department of Medicine Information Technology, University of Washington Medical Center, Seattle, Washington, United States of America; Virginia Commonwealth University School of Medicine, UNITED STATES OF AMERICA

## Abstract

**Background:**

The National Health Interview Survey (NHIS), a nationwide in-person and telephone survey of the civilian non-institutionalized population, is used to measure progress towards Healthy People objectives on cancer screening. Survey reports have been compared to medical record data to assess validity of these questions; however, few studies have assessed reliability, or response consistency over time. We assessed the test-retest reliability of questions on cervical, colorectal, breast and lung cancer screening from the 2021 and 2022 NHIS.

**Methods:**

We surveyed 1,770 adults ages 18 + years with and without prior screening for breast, cervical, colorectal, and lung cancer from four U.S. health systems (three in Washington state and one in Michigan) to examine validity of the NHIS questions. Among people who completed the validity survey, we randomized individuals to receive an additional survey one month later (N = 944) and three months later (N = 822). We calculated Cohen’s Kappa, Gwet’s AC1, and simple concordance to assess reliability.

**Results:**

Among our study sample, 535 (56.7% of those assigned) to one month follow-up and 537 (65.0%) assigned to three month follow-up completed the reliability survey. Among these, 36 (6.6%) and 94 (17.6%) of those assigned to one and three month follow-up respectively were excluded from reliability analyses because they had additional screening tests after completing the validity survey. Cohen’s Kappa showed strong-to-moderate reliability for being up to date with mammography screening: 0.83 at one month and 0.78 at three months; reliability for other screening tests was considerably lower: 0.61 and 0.60 for cervical, 0.57 and 0.62 for colorectal, and 0.72 and 0.58 for lung and one and three months, respectively.

**Conclusions:**

We found strong-to-moderate reliability of responses recalling breast cancer screening at one and three months following an initial survey. Reliability may not be a major threat to self-reported cancer screening behavior.

## Introduction

Cancer was the second leading cause of death, after heart disease, in the United States (U.S.) in 2024 [1,2]. According to modeling done by the NCI Cancer Intervention and Surveillance Modeling Network (CISNET) program, an estimated 5.94 million cancer deaths were averted for breast, cervical, colorectal, lung, and prostate cancers combined between 1975 and 2020 [3]. Cancer screening efforts played an important role in averting some of these deaths. Routine cancer screenings can lead to prevention or earlier detection for some cancers, notably cervical, breast, colorectal, and lung cancers, before symptoms appear and when cancer is often most treatable [4].

For the past four decades, the U.S. Department of Health and Human Services (US DHHS) has developed measurable public health objectives every ten years, known as the Healthy People objectives. In Healthy People 2030, the objectives focus on promoting evidence-based cancer screening and prevention strategies [5]. Determining whether the US population meets these goals is a critical step in developing and modifying interventions to increase the appropriate use of cancer screenings [6]. To measure progress towards the Healthy People objectives, the US DHHS utilizes data from the National Health Interview Survey (NHIS), a nationwide in-person and telephone survey of the civilian non-institutionalized population conducted by the National Center for Health Statistics, Centers for Disease Control and Prevention (CDC), including cancer screening utilization [7–12].

Four prior studies have evaluated the reliability of cancer screening questions (not necessarily NHIS questions) with mixed results [13–16]. Reliability varied across these studies depending on the cancer screening site with studies showing slightly better reliability for questions about breast cancer screening than colorectal cancer screening. Vernon et al recognized that, “Qualitative data from focus groups showed that many adults do not know or recognize the names of CRCS [colorectal cancer screening] tests and that many were unable to distinguish between SIG [sigmoidoscopy] and COL [colonoscopy] or between home-based and office-based FOBT [fecal occult blood testing].” In our cognitive study of cervical cancer screening, we found similar problems with understanding specific cancer screening tests including a lack of understanding of human papilloma virus testing [17]. We are unaware of studies that have evaluated the reliability of cervical or lung cancer screening questions.

Using a modified version of the NHIS Cancer Control Supplement (CCS), we evaluated reliability of cancer screening questions at four separate health care systems that differ in administrative structures and populations serviced. In this paper, we analyze and present data on the reliability of these questions at intervals of 1 and 3 months after their first survey. Along with the validity results of this study, published elsewhere [18], these reliability estimates will help assess whether the data from the NHIS presents an accurate reflection of the true cancer screening behavior of the respondents to the survey.

## Materials and methods

The overall study methods, published elsewhere, are summarized here [19]. We randomized 1,770 adults ages 18 + years to complete a web- or phone-based survey on cancer screening using a modified version of the NHIS CCS questions. The study settings included three health systems in Washington state: University of Washington (UW), Kaiser Permanente Washington (KPWA), and MultiCare Health System (MHS). A fourth setting in Michigan, Henry Ford Health (HFH), was later added to achieve further population diversity and to increase recruitment of participants eligible for lung cancer screenings. We obtained IRB approval to begin collection of electronic health record data on March 21, 2022. We recruited participants from June 1, 2022, through March 31, 2024.

### Design

**Fig 1:**
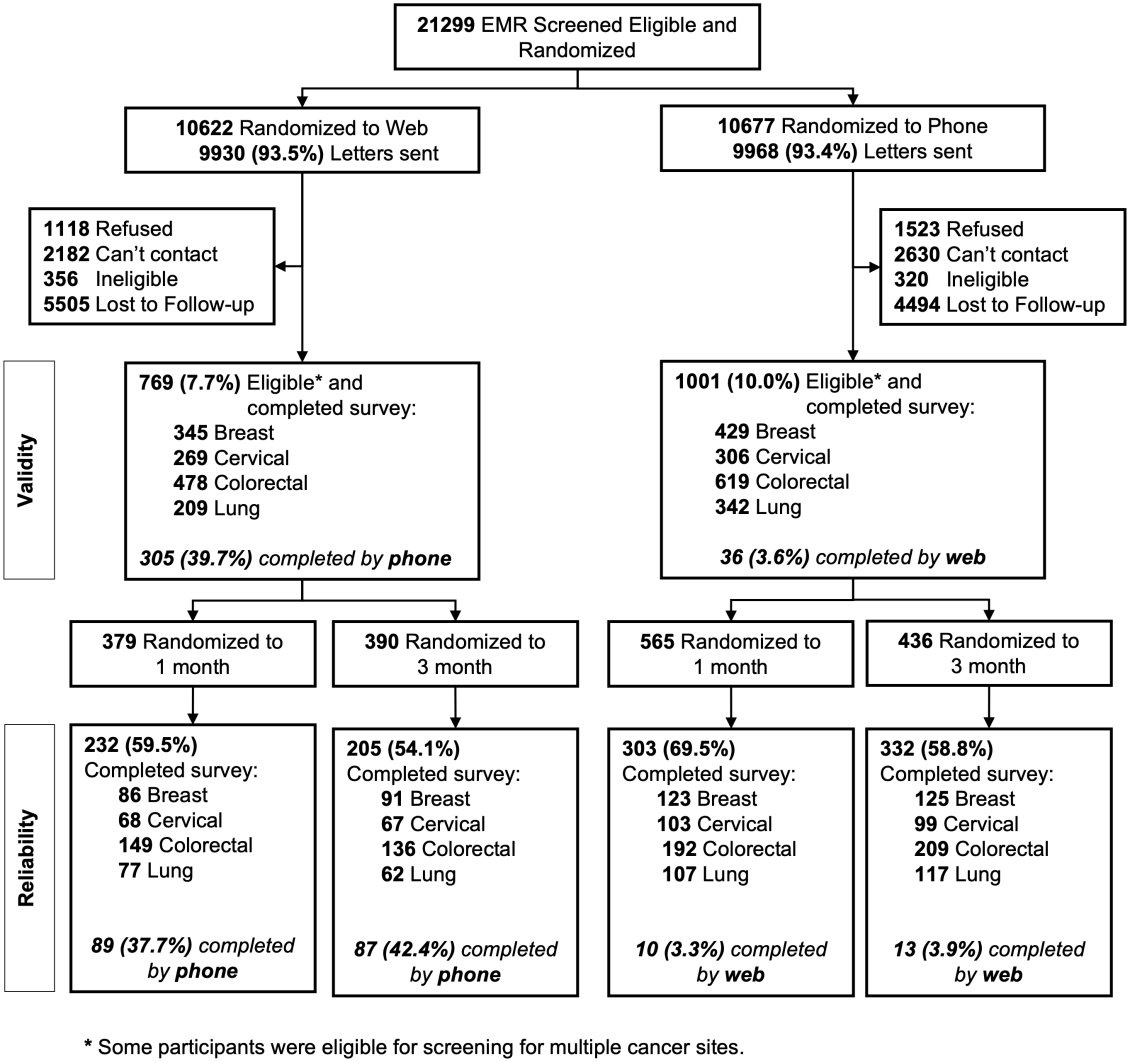
SIP screening and enrollment CONSORT diagram.

The randomized aspect of this study compared phone- vs. web-based survey questions about breast, colorectal, cervical and lung cancer screening to gold-standard data from electronic medical records (EMRs). To test whether the questions elicit reliable answers, we randomized all participants to receive an additional survey at either 1 or 3 months after their initial survey. We chose the 1-month time interval because it was sufficiently short so that we would expect little to no changes in the interim. The 3 month recall period corresponded to the literature in the field [16]. Figure 1 shows that of the 769 validity surveys completed with an initial assignment of web completion, 379 were randomized to 1 month follow up surveying, with 232 completions (59.4%), and an additional 390–3 month follow up surveying with 205 completions (54.1%). For those with initial assignment of phone completion, we had 1001 total interviews; 565 were randomized to 1 month, with 303 completions (69.5%), and an additional 436–3 months with 332 completions (58.8%) (Fig 1).

### Ethical approval

Recruitment, consent, and study procedures were reviewed and approved by the University of Washington Institutional Review Board (STUDY 12071). KPWA, and MHS sites ceded human subjects review to the University of Washington. Henry Ford Health obtained IRB approval from Henry Ford Health Institutional Review Board (STUDY 16261). Verbal and digital consent is documented for each participant in our databases.

### Setting

The three Washington health systems recruited people eligible for breast, colorectal, cervical, and lung cancer screening, whereas HFH only recruited people eligible for lung cancer screening, those participants eligible for additional cancer screening at the time of the survey were asked to complete those screening questions.

### Stratification and Randomization

We extracted data on prior cancer screening from electronic medical records (EMRs). Participants were stratified by race and ethnicity with the goal of recruiting at least 25% Non-Hispanic Black participants and 25% Hispanic participants for the study population [19].

### Reliability assessments

We calculated three standard measures of reliability: simple agreement or concordance from the first to second survey (as a percentage), Cohen’s Kappa which adjusts for chance, and Gwet’s AC1, which also does an adjustment for chance [20,21]. Gwet’s AC1 is a statistical measure used to assess the agreement between two or more raters, calculating the probability of agreement between them beyond what would be expected by chance. This is similar to Cohen’s Kappa but with a slightly different interpretation, as it compares observed agreement with an expected disagreement rate rather than an expected agreement rate [22]. We assessed reliability by screening test and by sex. We also performed sensitivity analyses by web or phone survey mode.

We presented the proportion of respondents showing concordance by cancer and test type and assigned follow-up period and calculated an odds ratio comparing agreement between 1- and 3-month follow-up for each cancer and test type.

## Results

Table 1 shows the demographic composition of respondents. For these screening tests, women are eligible for all tests, while men are only candidates for colorectal and lung cancer screening, thus our sample included nearly 70% women. The age distribution skewed towards older ages because of eligibility criteria for screening. Our sample contains 59% White persons as we oversampled for participants in other racial categories. Our fourth site (HFH) only selected people whose EMR characterized as likely eligible for lung cancer screening. We did not see any substantial differences in the demographic characteristics of the 1-month vs. the 3-month survey respondents, with the exception that increasing time between validity and reliability surveys appeared to result in a greater decrease in responding among Black participants as compared with White participants, resulting in a higher proportion of White respondents for validity surveys administered at 3 months.

**Table 1:** Population characteristics by assigned follow-up period among those who completed reliability surveys.

	1 Month	3 Month	Overall
Age: 21–29 Years	11 (2.0%)	19 (3.5%)	30 (2.8%)
Age: 30–39 Years	26 (4.8%)	27 (5.0%)	53 (4.9%)
Age: 40–49 Years	32 (5.9%)	26 (4.8%)	58 (5.4%)
Age: 50–59 Years	84 (15.5%)	89 (16.6%)	173 (16.0%)
Age: 60–69 Years	242 (44.7%)	227 (42.3%)	469 (43.5%)
Age: 70 + Years	146 (27.0%)	149 (27.7%)	295 (27.4%)
Sex (At Birth): Female	356 (65.7%)	355 (66.1%)	711 (65.9%)
Sex (At Birth): Male	186 (34.3%)	182 (33.9%)	368 (34.1%)
Race: AIAN American Indian/Alaska Native	13 (2.4%)	7 (1.3%)	20 (1.9%)
Race: Asian	35 (6.5%)	41 (7.6%)	76 (7.0%)
Race: Black	118 (21.8%)	71 (13.2%)	189 (17.5%)
Race: NHPI Non-Hispanic Pacific Islander	5 (0.9%)	5 (0.9%)	10 (0.9%)
Race: White	312 (57.6%)	355 (66.1%)	667 (61.8%)
Race: Multiple/Other	57 (10.5%)	57 (10.6%)	114 (10.6%)
Race: Unknown	2 (0.4%)	1 (0.2%)	3 (0.3%)
Ethnicity: Hispanic	48 (8.9%)	56 (10.4%)	104 (9.6%)
Ethnicity: Non-Hispanic	494 (91.1%)	481 (89.6%)	975 (90.4%)
Smoking status (Never)	266 (49.1%)	283 (52.7%)	549 (50.9%)
Smoking status (Former)	144 (26.6%)	133 (24.8%)	277 (25.7%)
Smoking status (Current)	131 (24.2%)	121 (22.5%)	252 (23.4%)
Smoking status (Unknown)	1 (0.2%)	0 (0.0%)	1 (0.1%)

In Table 2, we examine the relationship between the survey responders at 1 and 3 months and whether they self-reported being up to date for screening to determine if there were any notable differences between these groups and screening reports.

**Table 2:** Percent up to date (UTD) by self-report in initial survey among those that responded to the reliability survey.

	N	Overall % up to date	1 month % up to date	3 month % up to date
Breast	434	79.49	77.31	81.65
Cervical	289	79.93	78.08	81.82
Colorectal	652	85.28	82.76	87.69
Lung	344	54.94	55.49	54.39

In Table 3, we observe generally high measures of test-retest reliability. Survey respondents reported similarly to the initial survey regardless of whether the questions about screening were asked 1 or 3 months later. While concordance was near or above 80% for surveys administered at 1 or 3 months in all cancer types, this was partly due to chance agreement resulting from the high proportion of participants reporting up-to-date screening. As Cohen’s Kappa and Gwet’s AC1 correct for chance agreement, these statistics tended to be lower. Reliability statistics did not seem to be markedly lower for the 3-month period compared to the 1-month period. While there appears to be a notable degradation in agreement for lung cancer, we also note the confidence intervals overlap.

**Table 3:** Reliability statistics stratified by both sex and follow-up period.

Breast (Female only)			
	**Gwet’s AC1**	**Kappa**	**Concordance**
1 Month	0.916 (0.866, 0.967)	0.829 (0.731, 0.927)	0.944 (0.902, 0.972)
3 Month	0.908 (0.855, 0.962)	0.779 (0.659, 0.898)	0.935 (0.889, 0.966)
**Cervical (Female only)**			
	**Gwet’s AC1**	**Kappa**	**Concordance**
1 Month	0.810 (0.719, 0.901)	0.609 (0.446, 0.773)	0.872 (0.806, 0.923)
3 Month	0.835 (0.748, 0.922)	0.596 (0.412, 0.780)	0.883 (0.814, 0.933)
**Colorectal**			
	**Gwet’s AC1**	**Kappa**	**Concordance**
Total, 1 Month	0.830 (0.773, 0.886)	0.568 (0.445, 0.691)	0.878 (0.836, 0.912)
Total, 3 Month	0.883 (0.837, 0.929)	0.623 (0.493, 0.753)	0.911 (0.873, 0.94)
Female, 1 Month	0.797 (0.720, 0.875)	0.516 (0.362, 0.669)	0.857 (0.801, 0.902)
Female, 3 Month	0.912 (0.862, 0.962)	0.672 (0.505, 0.838)	0.930 (0.884, 0.962)
Male, 1 Month	0.888 (0.811, 0.964)	0.679 (0.484, 0.874)	0.917 (0.848, 0.961)
Male, 3 Month	0.832 (0.739, 0.925)	0.560 (0.355, 0.764)	0.878 (0.804, 0.932)
**Lung**			
	**Gwet’s AC1**	**Kappa**	**Concordance**
Total,1 month	0.731 (0.619, 0.843)	0.717 (0.602, 0.832)	0.862 (0.795, 0.914)
Total, 3 month	0.588 (0.444, 0.733)	0.58 (0.436, 0.723)	0.792 (0.71, 0.859)
Female, 1 Month	0.807 (0.666, 0.949)	0.786 (0.635, 0.936)	0.899 (0.802, 0.958)
Female, 3 Month	0.639 (0.443, 0.836)	0.598 (0.396, 0.800)	0.810 (0.691, 0.898)
Male, 1 Month	0.661 (0.488, 0.834)	0.655 (0.484, 0.826)	0.829 (0.725, 0.906)
Male, 3 Month	0.549 (0.335, 0.763)	0.552 (0.349, 0.755)	0.774 (0.650, 0.871)

Table 3 presents reliability statistics by follow-up period, cancer type, and sex of the respondent. Generally, there are no consistent or material differences in the reliability statistics by 1- or 3-month response. However, for lung cancer screening, across all measures of reliability, there seems a trend for men to have lower reliability statistics than women, though the confidence intervals of the reliability measures by sex overlap.

In Table 4, we present reliability statistics by assigned mode, either to phone-based or web-based interviews. The confidence intervals of the reliability statistics overlap with no consistent pattern of reliability that favors one mode of survey administration. We present these data by 1-month and 3-month intervals in S1 Table.

**Table 4:** Reliability statistics by assigned modality.

Breast (Female only)			
	**Gwet’s AC1**	**Kappa**	**Concordance**
Web	0.953 (0.911, 0.995)	0.895 (0.805, 0.985)	0.968 (0.926, 0.989)
Phone	0.885 (0.830, 0.939)	0.746 (0.636, 0.857)	0.921 (0.878, 0.952)
**Cervical (Female only)**			
	**Gwet’s AC1**	**Kappa**	**Concordance**
Web	0.797 (0.687, 0.908)	0.611 (0.428, 0.795)	0.867 (0.786, 0.925)
Phone	0.837 (0.761, 0.914)	0.597 (0.433, 0.760)	0.884 (0.825, 0.929)
**Colorectal**			
	**Gwet’s AC1**	**Kappa**	**Concordance**
Web	0.870 (0.816, 0.924)	0.619 (0.480, 0.758)	0.903 (0.859, 0.937)
Phone	0.847 (0.798, 0.896)	0.577 (0.462, 0.693)	0.888 (0.851, 0.918)
**Lung**			
	**Gwet’s AC1**	**Kappa**	**Concordance**
Web	0.716 (0.578, 0.854)	0.696 (0.554, 0.837)	0.853 (0.769, 0.915)
Phone	0.635 (0.516, 0.753)	0.627 (0.509, 0.746)	0.815 (0.748, 0.871)

Table 5 shows concordance by survey time period and cancer type and screening type. This also shows that agreement does not differ between 1-month and 3-month surveys for any cancer type.

**Table 5:** Proportion with concordance by assigned follow-up period and their odds ratios.

	1 Month	3 Month	OR* (95% CI)
Breast	0.944 (0.902, 0.972)	0.935 (0.889, 0.966)	1.167 (0.502, 2.713)
Cervical	0.872 (0.806, 0.923)	0.883 (0.814, 0.933)	0.907 (0.437, 1.885)
Colonoscopy	0.933 (0.899, 0.959)	0.928 (0.894, 0.954)	1.084 (0.583, 2.018)
Lung	0.862 (0.795, 0.914)	0.792 (0.71, 0.859)	1.641 (0.866, 3.112)
Pap smear	0.768 (0.689, 0.834)	0.769 (0.687, 0.839)	0.991 (0.564, 1.742)
HPV test	0.812 (0.739, 0.873)	0.772 (0.692, 0.84)	1.279 (0.717, 2.283)
Colorectal	0.878 (0.836, 0.912)	0.911 (0.873, 0.94)	0.705 (0.419, 1.188)
FOBT/FIT	0.946 (0.914, 0.969)	0.907 (0.867, 0.939)	1.791 (0.939, 3.414)
DNA-FIT	0.952 (0.919, 0.974)	0.947 (0.913, 0.97)	1.119 (0.522, 2.398)

*Comparing 1-month to 3-month assigned follow-up

## Discussion

According to the CDC, accurate cancer screening data are necessary for effective public health surveillance that informs resource allocation, program evaluation, and policy decisions aimed at cancer control [23].

Few studies report on the reliability of cancer screening self-reports. We add to that literature, and are generally in agreement with prior work such as that by Bradbury et al. and Vernon et al [14,16]. We noted higher reliability between measures at 1 month and limited degradation in respondent recall of prior screening examinations.

Prior work suggests that for health studies, Cohen’s Kappa should have a more rigorous recommendation than previously thought and we use the ranges from McHugh to describe the strength of our findings [24]. For our reliability statistics we report by cancer type. We note that for breast cancer, the reliability statistics are strong (.80−.90) to moderate (.60−.79), whereas for the other three cancers, they tend to fall in the moderate to weak (.40−.59) categories, recognizing that the simple agreement (concordance) statistics are quite strong. Regarding variations between modalities, our findings are consistent with Vernon et al. (2008) [16], who reported only modest mode-of-administration effects on reliability of colorectal screening questions across mail, telephone, and face-to-face administration. Given the NHIS’s accelerating shift toward mixed-mode (telephone and household) data collection (Sample Adult interviews in 2021 were 62.8% telephone-administered), the absence of a meaningful mode effect in our data is reassuring for the comparability of reliability across NHIS administration modes over time.

We also considered how prevalence imbalance may affect agreement statistics by considering the phenomenon, known as the prevalence paradox or kappa paradox [25–27]. The proportion of agreement will tend to approach 100% as the proportion of ratings becomes more unbalanced, i.e., the proportion of ratings approaches 100% in one category and 0% in the other, even in the absence of any association between the two raters. This can be misleading if a high proportion of ratings fall into one category or the other. Cohen’s Kappa accounts for this by comparing the observed rate of agreement to the rate of agreement which would be expected if the two ratings were unrelated. Therefore, the interpretation of Cohen’s Kappa is the same regardless of the prevalence of each rating, ranging from a value of zero indicating no association between reviewers and one indicating perfect agreement. Gwet’s AC compares the rate of agreement to the expected rate of disagreement and will tend to increase when the proportion of ratings approaches 100% in one category, causing the expected rate of disagreement to approach zero, even if there is no association between reviewers. Therefore, the interpretation of Gwet’s AC changes as the prevalence of each rating changes [28].

### Limitations

The context for evaluating these questions that are used in the CDC National Center for Health Statistics’ (NCHS) National Health Interview Survey is one where the primary approach has historically been in the household. The COVID pandemic dramatically changed that practice. For example, in 2021, 62.8% of the Sample Adult interviews and 61.4% of the Sample Child interviews were conducted at least partially by telephone [29]. While the intent of the NCHS is to return to household interviewing, it is likely at least a portion of the survey will be done by phone. Thus, there may be some loss of generalizability from our study to the NHIS. However, while our mode mix (telephone and web, no in-person) differs from the historical NHIS mode mix, it is increasingly similar to the current NHIS administration, which is approximately 60% telephone-based.

Likely more important may be our response rates. Whereas NHIS has experienced at least a 50% response rate to its survey requests, our study had only an 8.9% response rate. Our response rates merit careful interpretation. The 8.9% figure cited above refers to recruitment into the initial (validity) survey from health-system membership rolls and is the relevant denominator for evaluating the representativeness of our sample composition. The relevant denominator for evaluating non-response bias in our reliability estimates, however, is the response rate to the randomized follow-up among those who already completed the validity survey: 56.7% at 1 month and 65.0% at 3 months. Thus, our sample may be biased if survey responders differ from non-responders. This potential bias may be further exacerbated by those who agreed to do a second survey, at either 1 or 3 months, where our retention is quite respectable. It is possible, then, that our data and conclusions might not be generalizable to a more representative population

It is quite possible that this sample overrepresents individuals who are more engaged with healthcare and more likely to remember their screening attendance, potentially inflating reliability estimates. That would suggest that our reliability estimates may be an overestimate of what one would find if any study were able to universally reach a population and ask them screening questions at spaced intervals. Such studies are exceedingly uncommon but would help address questions around generalizability of results in the future.

Additionally, the exclusion of participants who obtained screening between surveys may further bias results by restricting the analysis to a more stable subgroup, thereby overstating agreement.

We also note that we performed our surveys starting in 2022 and ending in 2024. This began during the pandemic when there was a well-documented decrease in cancer screening [30–31]. There is no a priori reason to believe that recalling cancer screening would be more or less accurate with the intervening pandemic. It is, in fact, possible that agreement might be higher because going for screening, certainly early in the pandemic, might have been very salient because clinical contact was so proscribed, particularly early in 2020.

Finally, the majority of our sample is derived from Washington state. Because this area of the country is low in minority representation, we compensated by oversampling non-Hispanic Black and Hispanic participants where we had race and ethnicity information available. Even with that approach, it bears repeating that most of our sample comes from one region of the country. We believe that our findings are robust, however, as in other cases of our limitations, our results across sites are quite similar to each other, suggesting a lack of bias in our predominantly northwest dwelling participants. Based on the overall consistency across test types and the various agreement statistics we examined.

## Conclusion

In a study of four health systems, we asked respondents to report their cancer screening experience at intervals of 1 or 3 months after an initial survey and found moderate to strong reliability in responses for breast cancer screening. We also saw little to no diminution of recall from 1 to 3 months in reliability statistics except in the case of lung cancer screening. Overall, we conclude that reliability may not be a major threat to the interpretation of results from asking about cancer screening behavior; however, as our response rates for this study were quite low, we would encourage further study of reliability of cancer screening questions to consider if these are the best ways for the US to monitor cancer screening behavior

## Supporting information

S1 Table Reliability statistics by assigned modality and follow-up. (DOCX)
