# Supplementary material for: Reliability of cancer screening questions from the National Health Interview Survey
Source: PLoS One. 2026 Jul 1;21(7):e0352356. doi: 10.1371/journal.pone.0352356 (PMC13322553; doi:10.1371/journal.pone.0352356)
Supplement: S1 Table — (DOCX) [file pone.0352356.s001.docx]

# S1 Table. Reliability statistics by assigned modality and follow-up

| Reliability statistics by assigned modality, 1 month | | | |
| --- | --- | --- | --- |
| Breast (Female only) | | | |
|  | Gwet’s AC1 | Kappa | Concordance |
| Web | 0.943 (0.877, 1.000) | 0.885 (0.758, 1.000) | 0.962 (0.893, 0.992) |
| Phone | 0.899 (0.826, 0.971) | 0.790 (0.652, 0.929) | 0.932 (0.870, 0.970) |

Cervical (Female only)

|  | Gwet’s AC1 | Kappa | Concordance |
| --- | --- | --- | --- |
| Web | 0.820 (0.678, 0.962) | 0.592 (0.322, 0.861) | 0.875 (0.759, 0.948) |
| Phone | 0.804 (0.683, 0.925) | 0.620 (0.416, 0.824) | 0.871 (0.780, 0.934) |

Colorectal

|  | Gwet’s AC1 | Kappa | Concordance |
| --- | --- | --- | --- |
| Web | 0.843 (0.760, 0.926) | 0.599 (0.416, 0.782) | 0.887 (0.821, 0.935) |
| Phone | 0.820 (0.742, 0.897) | 0.546 (0.382, 0.709) | 0.871 (0.812, 0.916) |

Lung

|  | Gwet’s AC1 | Kappa | Concordance |
| --- | --- | --- | --- |
| Web | 0.868 (0.739, 0.997) | 0.865 (0.738, 0.993) | 0.933 (0.838, 0.982) |
| Phone | 0.638 (0.470, 0.806) | 0.608 (0.436, 0.780) | 0.812 (0.712, 0.888) |

# Reliability statistics by assigned modality, 3 months

| Breast (Female only) | | | |
| --- | --- | --- | --- |
|  | Gwet’s AC1 | Kappa | Concordance |
| Web | 0.963 (0.909, 1.000) | 0.907 (0.781, 1.000) | 0.973 (0.907, 0.997) |
| Phone | 0.871 (0.787, 0.954) | 0.695 (0.518, 0.871) | 0.909 (0.839, 0.956) |

Cervical (Female only)

|  | Gwet’s AC1 | Kappa | Concordance |
| --- | --- | --- | --- |
| Web | 0.770 (0.591, 0.948) | 0.624 (0.372, 0.877) | 0.857 (0.728, 0.941) |
| Phone | 0.870 (0.774, 0.965) | 0.543 (0.263, 0.822) | 0.899 (0.810, 0.955) |

Colorectal

|  | Gwet’s AC1 | Kappa | Concordance |
| --- | --- | --- | --- |
| Web | 0.900 (0.831, 0.968) | 0.645 (0.431, 0.859) | 0.922 (0.857, 0.964) |
| Phone | 0.872 (0.811, 0.934) | 0.611 (0.448, 0.774) | 0.904 (0.852, 0.942) |

Lung

|  | Gwet’s AC1 | Kappa | Concordance |
| --- | --- | --- | --- |
| Web | 0.522 (0.245, 0.799) | 0.421 (0.134, 0.708) | 0.738 (0.580, 0.861) |
| Phone | 0.639 (0.469, 0.808) | 0.639 (0.473, 0.804) | 0.819 (0.720, 0.895) |
