# Supplementary material for: Reliability of cancer screening questions from the National Health Interview Survey
Source: PLoS One. 2026 Jul 1;21(7):e0352356. doi: 10.1371/journal.pone.0352356 (PMC13322553; doi:10.1371/journal.pone.0352356)
Supplement: Peer review history

# Peer review: Decision Letter 0

**DOI:** 10.1371/journal.pone.0352356.r001

**Authors:** David Zhu

22 Apr 2026

PONE-D-26-01649

Reliability of Cancer Screening Questions from the National Health Interview Survey

PLOS One

Dear Dr. Kessler,

Thank you for submitting your manuscript to PLOS ONE. After careful consideration, we feel that it has merit but does not fully meet PLOS ONE’s publication criteria as it currently stands. Therefore, we invite you to submit a revised version of the manuscript that addresses the points raised during the review process.

==============================

Both reviewers noted the strong motivation and clear writing. However, I agree with Reviewer 1 that the low response rate raises concerns about generalizability. I encourage the authors to consider a sensitivity analysis that broadens the analytic sample and/or an additional analysis to assess and address potential nonresponse bias.

==============================

Please submit your revised manuscript by Jun 06 2026 11:59PM. If you will need more time than this to complete your revisions, please reply to this message or contact the journal office at plosone@plos.org. When you're ready to submit your revision, log on to https://www.editorialmanager.com/pone/ and select the 'Submissions Needing Revision' folder to locate your manuscript file.

Please include the following items when submitting your revised manuscript:

- A letter that responds to each point raised by the academic editor and reviewer(s). You should upload this letter as a separate file labeled 'Response to Reviewers'.
- A marked-up copy of your manuscript that highlights changes made to the original version. You should upload this as a separate file labeled 'Revised Manuscript with Track Changes'.
- An unmarked version of your revised paper without tracked changes. You should upload this as a separate file labeled 'Manuscript'.

If you would like to make changes to your financial disclosure, please include your updated statement in your cover letter. Guidelines for resubmitting your figure files are available below the reviewer comments at the end of this letter.

If applicable, we recommend that you deposit your laboratory protocols in protocols.io to enhance the reproducibility of your results. Protocols.io assigns your protocol its own identifier (DOI) so that it can be cited independently in the future. For instructions see: https://journals.plos.org/plosone/s/submission-guidelines#loc-laboratory-protocols. Additionally, PLOS ONE offers an option for publishing peer-reviewed Lab Protocol articles, which describe protocols hosted on protocols.io. Read more information on sharing protocols at https://plos.org/protocols?utm_medium=editorial-email&utm_source=authorletters&utm_campaign=protocols.

As the corresponding author, your ORCID iD is verified in the submission system and will appear in the published article. PLOS supports the use of ORCID, and we encourage all coauthors to register for an ORCID iD and use it as well. Please encourage your coauthors to verify their ORCID iD within the submission system before final acceptance, as unverified ORCID iDs will not appear in the published article. *Only*  the individual author can complete the verification step; PLOS staff *cannot*  verify ORCID iDs on behalf of authors.

We look forward to receiving your revised manuscript.

Kind regards,

David T. Zhu

Academic Editor

PLOS One

Journal Requirements:

When submitting your revision, we need you to address these additional requirements.

1. Please ensure that your manuscript meets PLOS ONE's style requirements, including those for file naming. The PLOS ONE style templates can be found at

https://journals.plos.org/plosone/s/file?id=wjVg/PLOSOne_formatting_sample_main_body.pdf and

https://journals.plos.org/plosone/s/file?id=ba62/PLOSOne_formatting_sample_title_authors_affiliations.pdf.

2. Thank you for stating the following financial disclosure:

“In 2020, the Centers for Disease Control issued a cooperative agreement to the University of Washington (Coop Agreement #5 U48DP006398-02-00) to assess the validity and reliability of the redesigned questions on cervical, colorectal, breast, and lung cancer screening from the 2020-2022 National Health Interview Survey. Henry Ford received funds from ICF International Inc. (Subcontract GS-00F-010CA/HHSN261201700003B/75N91022F00002/TO9) “

Please state what role the funders took in the study.  If the funders had no role, please state: "The funders had no role in study design, data collection and analysis, decision to publish, or preparation of the manuscript."

If this statement is not correct you must amend it as needed.

Please include this amended Role of Funder statement in your cover letter; we will change the online submission form on your behalf.

3.We note that you have indicated that there are restrictions to data sharing for this study. For studies involving human research participant data or other sensitive data, we encourage authors to share de-identified or anonymized data. However, when data cannot be publicly shared for ethical reasons, we allow authors to make their data sets available upon request. For information on unacceptable data access restrictions, please see http://journals.plos.org/plosone/s/data-availability#loc-unacceptable-data-access-restrictions.

Before we proceed with your manuscript, please address the following prompts:

a) If there are ethical or legal restrictions on sharing a de-identified data set, please explain them in detail (e.g., data contain potentially identifying or sensitive patient information, data are owned by a third-party organization, etc.) and who has imposed them (e.g., a Research Ethics Committee or Institutional Review Board, etc.). Please also provide contact information for a data access committee, ethics committee, or other institutional body to which data requests may be sent.

b) If there are no restrictions, please upload the minimal anonymized data set necessary to replicate your study findings to a stable, public repository and provide us with the relevant URLs, DOIs, or accession numbers. Please see http://www.bmj.com/content/340/bmj.c181.long for guidelines on how to de-identify and prepare clinical data for publication. For a list of recommended repositories, please see https://journals.plos.org/plosone/s/recommended-repositories. You also have the option of uploading the data as Supporting Information files, but we would recommend depositing data directly to a data repository if possible.

Please update your Data Availability statement in the submission form accordingly.

4.Please be informed that funding information should not appear in the Acknowledgments section or other areas of your manuscript. We will only publish funding information present in the Funding Statement section of the online submission form. Please remove any funding-related text from the manuscript.

5. Please include captions for your Supporting Information files at the end of your manuscript, and update any in-text citations to match accordingly. Please see our Supporting Information guidelines for more information: http://journals.plos.org/plosone/s/supporting-information.

6. If the reviewer comments include a recommendation to cite specific previously published works, please review and evaluate these publications to determine whether they are relevant and should be cited. There is no requirement to cite these works unless the editor has indicated otherwise.

Additional Editor Comments (if provided):

Both reviewers noted the strong motivation and clear writing. However, I agree with Reviewer 1 that the low response rate raises concerns about generalizability. I encourage the authors to consider a sensitivity analysis that broadens the analytic sample and/or an additional analysis to assess and address potential nonresponse bias.

[Note: HTML markup is below. Please do not edit.]

Reviewers' comments:

Reviewer's Responses to Questions

**Comments to the Author**

1. Is the manuscript technically sound, and do the data support the conclusions?

The manuscript must describe a technically sound piece of scientific research with data that supports the conclusions. Experiments must have been conducted rigorously, with appropriate controls, replication, and sample sizes. The conclusions must be drawn appropriately based on the data presented.

Reviewer #1: Partly

Reviewer #2: Yes

**********

2. Has the statistical analysis been performed appropriately and rigorously?

Reviewer #1: Yes

Reviewer #2: Yes

**********

3. Have the authors made all data underlying the findings in their manuscript fully available?

The PLOS Data policy requires authors to make all data underlying the findings described in their manuscript fully available without restriction, with rare exception (please refer to the Data Availability Statement in the manuscript PDF file). The data should be provided as part of the manuscript or its supporting information, or deposited to a public repository. For example, in addition to summary statistics, the data points behind means, medians and variance measures should be available. If there are restrictions on publicly sharing data—e.g. participant privacy or use of data from a third party—those must be specified.

Reviewer #1: Yes

Reviewer #2: Yes

**********

4. Is the manuscript presented in an intelligible fashion and written in standard English?

PLOS ONE does not copyedit accepted manuscripts, so the language in submitted articles must be clear, correct, and unambiguous. Any typographical or grammatical errors should be corrected at revision, so please note any specific errors here.

Reviewer #1: Yes

Reviewer #2: Yes

**********

5. Review Comments to the Author

Please use the space provided to explain your answers to the questions above. You may also include additional comments for the author, including concerns about dual publication, research ethics, or publication ethics. (Please upload your review as an attachment if it exceeds 20,000 characters)

Reviewer #1: This manuscript addresses an important and policy-relevant question regarding the test–retest reliability of NHIS cancer screening measures. The study is well-motivated, and the use of multiple agreement statistics, including Cohen’s Kappa and Gwet’s AC1, is appropriate and strengthens the methodological approach. The randomized assignment to 1- versus 3-month follow-up periods is also a notable strength.

However, there are several important limitations that weaken the strength of the conclusions. Most notably, the very low response rate (8.9%) raises substantial concerns about non-response bias and limits the generalizability of the findings. The analytic sample likely overrepresents individuals who are more engaged with healthcare and more likely to accurately report screening behaviors, potentially inflating reliability estimates. Additionally, the exclusion of participants who received screening between surveys may further bias results by restricting the analysis to a more stable subgroup, thereby overstating agreement.

While the manuscript acknowledges some of these limitations, their implications are not fully incorporated into the interpretation of results. The conclusion that reliability is “not a major threat” appears somewhat overstated given the moderate agreement levels observed (e.g., Kappa values in the 0.57–0.62 range for some cancers) and the potential biases in the sample. A more cautious interpretation is warranted.

Further, the manuscript would benefit from additional analyses to strengthen its claims. These include (1) assessing and, if possible, adjusting for non-response bias, (2) conducting sensitivity analyses that include participants with intervening screenings, (3) examining potential survey mode effects (phone vs. web), and (4) providing more detailed discussion of how prevalence imbalance may affect agreement statistics.

Overall, this is a well-conceived study with a solid methodological foundation, but important limitations related to sample representativeness and interpretation need to be addressed. With additional analyses and a more tempered discussion of findings, the manuscript could make a valuable contribution to the literature.

My recommendation is for major revisions because the study is not fatally flawed, but core validity concerns are unresolved, key analyses are missing, and the core conclusions are overstated.

Reviewer #2: Dear authors,

Main goal of tha paper is clear, whole manuscript is up to a point and easy to read, I find the limitations section particularly strong.

Minor suggestions are:

1. check sentence on line 232: “It is possible, then, that our data and conclusions might not reflect would be found by …”

2. check Figure 1, it contains lines on diagram outside the scheme with words (horizontally spaced: reliablelit), which are on top of diagram numbers (Randomized to 3 month)

**********

6. PLOS authors have the option to publish the peer review history of their article (what does this mean?). If published, this will include your full peer review and any attached files.

If you choose “no”, your identity will remain anonymous but your review may still be made public.

**Do you want your identity to be public for this peer review?**  For information about this choice, including consent withdrawal, please see our Privacy Policy.

Reviewer #1: No

Reviewer #2: No

**********

[NOTE: If reviewer comments were submitted as an attachment file, they will be attached to this email and accessible via the submission site. Please log into your account, locate the manuscript record, and check for the action link "View Attachments". If this link does not appear, there are no attachment files.]

To ensure your figures meet our technical requirements, please review our figure guidelines: https://journals.plos.org/plosone/s/figures

You may also use PLOS’s free figure tool, NAAS, to help you prepare publication quality figures: https://journals.plos.org/plosone/s/figures#loc-tools-for-figure-preparation.

NAAS will assess whether your figures meet our technical requirements by comparing each figure against our figure specifications.

# Author comment: Author response to Decision Letter 1

**DOI:** 10.1371/journal.pone.0352356.r002

4 Jun 2026

We address the comments as follows:

Data availability: You asked, “a) If there are ethical or legal restrictions on sharing a de-identified data set, please explain them in detail (e.g., data contain potentially identifying or sensitive patient information, data are owned by a third-party organization, etc.) and who has imposed them (e.g., a Research Ethics Committee or Institutional Review Board, etc.). Please also provide contact information for a data access committee, ethics committee, or other institutional body to which data requests may be sent.”

Our main analysis dataset is available on request per our data availability statement. We only strip out identifying information as specified in the manuscript. However, we do not make the cognitive data available and now state the following in the manuscript, “Because of the difficulty of deidentifying these cognitive data, we have not made these available.”

Unfortunately, we do not have the funding to place our deidentified dataset in a public repository. Our funding from CDC ended last year and they do not have funds to support such an effort.

Per this comment, we have removed the funding information from the manuscript and now included it in the online submission.

4.Please be informed that funding information should not appear in the Acknowledgments section or other areas of your manuscript. We will only publish funding information present in the Funding Statement section of the online submission form. Please remove any funding-related text from the manuscript.

Also, you asked about the role the funders took in the study. We have done so and included this in our manuscript as follows. We are grateful that you have indicated your willingness to change the online submission form on our behalf.

The funders, staff of the Centers for Disease Control and Prevention and the National Cancer Institute who are also involved in the area of cancer screening surveillance, reviewed our study protocols and had substantive comments on our manuscript. They did not have a role in decisions to publish (though they did encourage us to publish) and they did not have final say in the manuscript, its analysis, or conclusions.

We have included captions per:

5. Please include captions for your Supporting Information files at the end of your manuscript, and update any in-text citations to match accordingly. Please see our Supporting Information guidelines for more information: http://journals.plos.org/plosone/s/supporting-information.

This did not apply to this manuscript:

6. If the reviewer comments include a recommendation to cite specific previously published works, please review and evaluate these publications to determine whether they are relevant and should be cited. There is no requirement to cite these works unless the editor has indicated otherwise.

We did address this comment about response rates:

Both reviewers noted the strong motivation and clear writing. However, I agree with Reviewer 1 that the low response rate raises concerns about generalizability. I encourage the authors to consider a sensitivity analysis that broadens the analytic sample and/or an additional analysis to assess and address potential nonresponse bias.

Our response can be found in the limitation section:

Likely more important may be our response rates. Whereas NHIS has experienced at least a 50% response rate to its survey requests, our study had only an 8.9% response rate. Our response rates merit careful interpretation. The 8.9% figure cited above refers to recruitment into the initial (validity) survey from health-system membership rolls and is the relevant denominator for evaluating the representativeness of our sample composition. The relevant denominator for evaluating non-response bias in our reliability estimates, however, is the response rate to the randomized follow-up among those who already completed the validity survey: 56.7% at 1 month and 65.0% at 3 months. Thus, our sample may be biased if survey responders differ from non-responders. This potential bias may be further exacerbated by those who agreed to do a second survey, at either 1 or 3 months, where our retention is quite respectable. However, we also note that reliability is a within-person property and is therefore less vulnerable to non-response bias than prevalence or validity would be: differential response would bias reliability only if non-responders were systematically more (or less) consistent in their own self-reports across occasions, a property for which we are unaware of any empirical evidence. It is possible, though, that our data and conclusions might not be generalizable to a more representative population.

Here are the associated reviewer comments that we feel we have addressed above:

However, there are several important limitations that weaken the strength of the conclusions. Most notably, the very low response rate (8.9%) raises substantial concerns about non-response bias and limits the generalizability of the findings. The analytic sample likely overrepresents individuals who are more engaged with healthcare and more likely to accurately report screening behaviors, potentially inflating reliability estimates. Additionally, the exclusion of participants who received screening between surveys may further bias results by restricting the analysis to a more stable subgroup, thereby overstating agreement.

While the manuscript acknowledges some of these limitations, their implications are not fully incorporated into the interpretation of results. The conclusion that reliability is “not a major threat” appears somewhat overstated given the moderate agreement levels observed (e.g., Kappa values in the 0.57–0.62 range for some cancers) and the potential biases in the sample. A more cautious interpretation is warranted.

With respect to the following comment, we have commented on non-response bias, added sensitivity analyses and data on survey mode, and added a comment on prevalence imbalance.

Further, the manuscript would benefit from additional analyses to strengthen its claims. These include (1) assessing and, if possible, adjusting for non-response bias, (2) conducting sensitivity analyses that include participants with intervening screenings, (3) examining potential survey mode effects (phone vs. web), and (4) providing more detailed discussion of how prevalence imbalance may affect agreement statistics.

We have addressed the minor comments from reviewer #2.

Reviewer #2: Dear authors,

Minor suggestions are:

1. check sentence on line 232: “It is possible, then, that our data and conclusions might not reflect would be found by …”

This has been changed to: It is possible, then, that our data and conclusions might not be generalizable to a more representative population

2. check Figure 1, it contains lines on diagram outside the scheme with words (horizontally spaced: reliablelit), which are on top of diagram numbers (Randomized to 3 month)

We have supplied a new TIFF figure that fixes this problem.

# Editor report: Decision Letter 1

**DOI:** 10.1371/journal.pone.0352356.r003

**Authors:** David Zhu, David Zhu

9 Jun 2026

Reliability of Cancer Screening Questions from the National Health Interview Survey

PONE-D-26-01649R1

Dear Dr. Kessler,

We’re pleased to inform you that your manuscript has been judged scientifically suitable for publication and will be formally accepted for publication once it meets all outstanding technical requirements.

Within one week, you’ll receive an e-mail detailing the required amendments. When these have been addressed, you’ll receive a formal acceptance letter and your manuscript will be scheduled for publication.

An invoice will be generated when your article is formally accepted. Please note, if your institution has a publishing partnership with PLOS and your article meets the relevant criteria, all or part of your publication costs will be covered. Please make sure your user information is up-to-date by logging into Editorial Manager at Editorial Manager® and clicking the ‘Update My Information' link at the top of the page. For questions related to billing, please contact billing support.

If your institution or institutions have a press office, please notify them about your upcoming paper to help maximize its impact. If they’ll be preparing press materials, please inform our press team as soon as possible -- no later than 48 hours after receiving the formal acceptance. Your manuscript will remain under strict press embargo until 2 pm Eastern Time on the date of publication. For more information, please contact onepress@plos.org.

Kind regards,

David T. Zhu

Academic Editor

PLOS One

Additional Editor Comments (optional):

Reviewers' comments:

# Editor report: Acceptance letter

**DOI:** 10.1371/journal.pone.0352356.r004

**Authors:** David Zhu, David Zhu

PONE-D-26-01649R1

PLOS One

Dear Dr. Kessler,

I'm pleased to inform you that your manuscript has been deemed suitable for publication in PLOS One. Congratulations! Your manuscript is now being handed over to our production team.

At this stage, our production department will prepare your paper for publication. This includes ensuring the following:

* All references, tables, and figures are properly cited

* All relevant supporting information is included in the manuscript submission,

* There are no issues that prevent the paper from being properly typeset

You will receive further instructions from the production team, including instructions on how to review your proof when it is ready. Please keep in mind that we are working through a large volume of accepted articles, so please give us a few days to review your paper and let you know the next and final steps.

Lastly, if your institution or institutions have a press office, please let them know about your upcoming paper now to help maximize its impact. If they'll be preparing press materials, please inform our press team within the next 48 hours. Your manuscript will remain under strict press embargo until 2 pm Eastern Time on the date of publication. For more information, please contact onepress@plos.org.

You will receive an invoice from PLOS for your publication fee after your manuscript has reached the completed accept phase. If you receive an email requesting payment before acceptance or for any other service, this may be a phishing scheme. Learn how to identify phishing emails and protect your accounts at https://explore.plos.org/phishing.

If we can help with anything else, please email us at customercare@plos.org.

Thank you for submitting your work to PLOS ONE and supporting open access.

Kind regards,

PLOS ONE Editorial Office Staff

on behalf of

Mr. David T. Zhu

Academic Editor

PLOS One
